# Seasonal Influenza Vaccination Among Saudi Children: Parental Barriers and Willingness to Vaccinate Their Children in the Makkah Region

**DOI:** 10.7759/cureus.38878

**Published:** 2023-05-11

**Authors:** Ibrahim Alharbi, Reem Alharthi, Shuaa Aljabri, Razan Alzhrani, Lujain Alzahrani, Saad Albagami

**Affiliations:** 1 Pediatrics, Hematology-Oncology, King Fahad Armed Forces Hospital, Jeddah, SAU; 2 Medicine and Surgery, Umm Al-Qura University, Makkah, SAU

**Keywords:** childhood vaccination, saudi arabia, makkah region, children, barriers, knowledge, parents, influenza vaccine, s: influenza virus

## Abstract

Introduction: The influenza virus produces everything from seasonal epidemics to unexpected pandemics, making its prevention and management a worldwide public health challenge. The main method to prevent and control seasonal influenza is vaccination. Children responded quite successfully to influenza vaccinations, particularly live vaccines. Despite the strong recommendations and effectiveness of seasonal influenza vaccinations in children, some parents still hesitate and refuse to give their children the shot.

Objective: This study, given the importance of knowing the factors that contribute to parents' refusal of influenza vaccine, also aims to assess parental barriers and willingness to vaccinate their children in the Makkah region of Saudi Arabia.

Methodology: This is a descriptive cross-sectional study conducted among Saudi parents in the Makkah region. For data collection, an online survey was conducted between December 1, 2022, and February 11, 2023.

Results: In total, 334 of the parents participated in our study. The result shows that there is a significant association between parents' gender and receiving the flu vaccine, with significantly more females (52.4%). Regarding the willingness to get the vaccine, the majority of the parents reported that they will get the vaccine and vaccinate their children, and the most common barrier that prevents parents from vaccinating their children is that they do not need it because they are healthy (21.48%). Furthermore, there is a strong relationship between educational level and knowledge about seasonal influenza vaccination; the majority of parents at each level of education have poor knowledge regarding influenza vaccines. In addition, nearly all of our participants (96.7%) believed the information provided by the Saudi Ministry of Health as well as the advice of their physicians.

Conclusion: This study highlights the need to increase awareness, educate the parents in the Makkah region about the importance of the influenza vaccine, and encourage them to vaccinate their children.

## Introduction

Influenza is a highly communicable viral disease that affects the upper and lower respiratory tracts and is caused by either the type A or type B influenza virus [1]. Each year, influenza viruses infect 20% of the world's population, causing three to five million serious illnesses and 290,000-650,000 deaths [2].

Influenza is characterized by symptoms of an upper respiratory tract infection, such as a runny nose, fever, cough, sore throat, and other symptoms like muscle pain, fatigue, and headache. However, some patients develop life-threatening complications [1].

It is generally diagnosed clinically and based on signs and symptoms. Vaccination is the primary preventive measure against influenza during the influenza season [3]. Priority is given to those who are considered high-risk groups: the elderly, those with underlying medical issues, kids, and pregnant women [4]. Moreover, the WHO recommends these groups for seasonal influenza immunization during the COVID-19 pandemic [5].

Due to the Hajj season, the Makkah region is more susceptible to seasonal influenza, which in turn accounts for the majority of hospital admissions [6]. This increases the pressure on the parents and healthcare systems; hence, it is essential to effectively identify and address the influencing factors on parental decision-making about vaccination and to assess their barriers and willingness to vaccinate in order to raise awareness and enhance the vaccination rates among the children of Makkah, therefore reducing the burden of the disease on both families and healthcare systems.

## Materials and methods

This is a descriptive cross-sectional study among Saudi parents in the Makkah region of Saudi Arabia. The study was conducted after getting ethical approval from the ethical committee of the Faculty of Medicine, Umm Al-Qura University, Saudi Arabia (IRB approval number: HAPO-02-K-012-2022-11-1238.)

The minimum sample size required for this study was calculated by the OpenEpi website to find the appropriate sample size. The population at which the study was aimed was around 2000 people, and in order for us to have a confidence level of 95% with a 5% error margin, we needed around 323 people.

For data collection, a semi-structured and self-administered online questionnaire was conducted between December 1, 2022, and February 11, 2023, through an Arabic online Google Form that was distributed using electronic devices to the targeted population.

The questionnaire contained four sections: consent form, exclusion questions, sociodemographic characteristics (age, gender, level of education, etc.), and assessment of barriers, willingness, and level of knowledge.

We included Saudi parents in the Makkah region of Saudi Arabia. We excluded non-Saudi parents or those who were from outside the Makkah region of Saudi Arabia. In total, 334 parents were included in our study.

For statistical analysis, we first extracted the data using Microsoft Office Excel 2013 into a spreadsheet for typographical error checking and coding. Then, IBM Corp.'s Statistical Package for the Social Sciences (SPSS) version 23 was used for data analysis. Descriptive statistics were obtained to summarize the data, synthesize it, and report the variables, in which numerical data are presented as the mean + standard deviation or as the median and range according to the type of distribution of each variable. For categorical variables, percentages and frequencies were used. The chi-squared test was utilized for the association between categorical variables. A p-value of 5% was considered statistically significant.

## Results

In total, 334 of the parents participated in our study. The number of mothers and fathers who participated in the study was 231 and 103, respectively, of whom the majority were women (69.2%), between the ages of 35 and 54 years (61.9%), and in the higher education category (bachelor’s degrees or higher) (77.5%). Exactly 158 (47.3%) participants had a job. Most of the participants had more than three children (125, 37.4%), 69 of them had three children (20.7%), 62 had two children (18.6%), and 78 (23.4%) had only one child. About 301 (90.1%) were vaccinated up-to-date; on the other hand, 18 (5.4%) were not, and 15 (4.5%) didn’t know. The majority of the participants (329, 98.5%) had heard about the influenza vaccine, while five (1.5%) had not (Table 1).

**Table 1:** The demographic data of the parents.

Variable	Category	Frequency (%)
Gender	Male	103 (30.8%)
	Female	231 (69.2%)
Age	18-24 years	34 (10.2%)
	25-34 years	69 (20.7%)
	35-44 years	113 (33.8%)
	45-54 years	94 (28.1%)
	55 and older	24 (7.2%)
Level of education	Primary + Intermediate education	19 (5.7%)
	High school education	56 (16.8%)
	Higher education bachelor master, doctoral	259 (77.5%)
Have a job	Yes	158 (47.3%)
	No	176 (52.7%)
Number of children	One child	78 (23.4%)
	Two children	62 (18.6%)
	Three children	69 (20.7%)
	More than three	125 (37.4%)
Up-to-date vaccination	Yes	301 (90.1%)
	No	18 (5.4%)
	I don’t know	15 (4.5%)
Heard about the flu vaccine	Yes	329 (98.5%)
	No	5 (1.5%)

When testing multiple factors that are associated with taking the influenza vaccine, it was found that there is a significant association between gender and receiving the vaccine, where significantly more women (121, 52.4%) reported that they did not receive the vaccine than men (2.28%) (p=0.000). The other factors show no significant association (Table 2).

**Table 2:** The association between the demographic data and receiving the influenza vaccine before.

Variable	Did the parents or their children receive the flu vaccine before?		p-value
	No (%)	Yes (%)	
Gender			
Male	29 (28.2%)	74 (71.8%)	0.000*
Female	121 (52.4%)	110 (47.6%)	
Age			
18-24 years	11 (32.4%)	23 (67.6%)	0.414
25-34 years	35 (50.7%)	34 (49.3%)	
35-44 years	54 (47.8%)	59 (52.2%)	
45-54 years	39 (41.5%)	55 (58.5%)	
55 and older	11 (45.8%)	13 (54.2%)	
Level of education			
Primary + Intermediate education	10 (52.6%)	9 (47.4%)	0.784
High school education	25 (44.6%)	31 (55.4%)	
Higher education- bachelor master, doctoral	115 (44.4%)	144 (55.6%)	
Have a job			
Yes	65 (41.1%)	93 (58.9%)	0.226
No	85 (48.3%)	91 (51.7%)	
Number of children			
One child	38 (48.7%)	40 (51.3%)	0.789
Two children	27 (43.5%)	35 (56.5%)	
Three children	28 (40.6%)	41 (59.4%)	
More than three	57 (45.6%)	68 (54.4%)	
Up-to-date vaccination			
Yes	132 (43.9%)	169 (56.1%)	0.007
No	14 (77.8%)	4 (22.2%)	
I don’t know	4 (26.7%)	11 (73.3%)	

**Figure 1:**
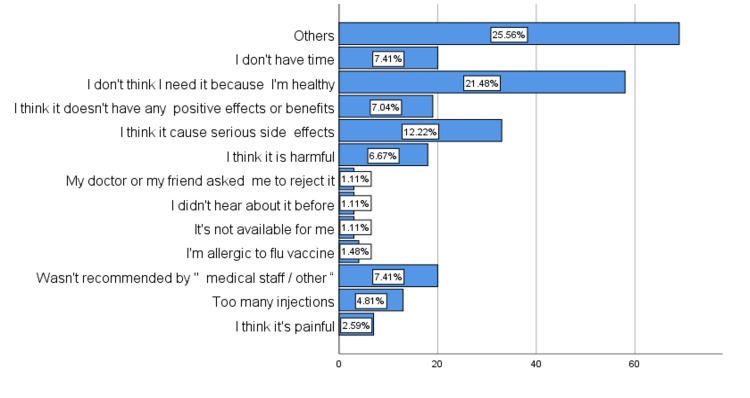
The barriers regarding receiving the influenza vaccine.

Regarding the willingness and barriers to getting the vaccine, 55.1% of the parents reported that they would get the vaccine and vaccinate their children, while 44.9% of them would not. It was found that the most common barriers were that they or their children do not need it because they are healthy (21.48%) and their thoughts about its potential side effects (12.22%), plus other barriers, as shown in Figure 1.

Table 3 shows the relationship between the parent's level of education and their level of knowledge about the influenza vaccine. The majority of parents at each level of education had poor knowledge regarding the influenza vaccine. Two hundred and twenty (84.9%) of the participants who had higher education (bachelor, master, or doctoral) had poor knowledge. While 17 (89.5%) of those who had primary or intermediate education had poor knowledge. None of them had good knowledge.

**Table 3:** The association between the level of education of the parents and their level of knowledge about the influenza vaccine.

Variable	Knowledge level about the influenza vaccination			p-value
	Good (%)	Moderate (%)	Poor (%)	
Level of education				
Primary + Intermediate education	0 (0.0%)	2 (10.5%)	17 (89.5%)	0.756
High school education	1 (1.8%)	8 (14.3%)	47 (83.9%)	
Higher education (bachelor master, doctoral)	2 (0.4%)	38 (14.7%)	220 (84.9%)	

Three hundred and sixteen (94.6%) parents were aware that the influenza vaccine is available and free in Saudi Arabia. The participants were quite familiar with awareness campaigns; 90.1% had heard of them. Nearly all of them (96.7%) believed the information provided by the Saudi Ministry of Health as well as the advice of their physicians (Table 4).

**Table 4:** Awareness of the parents about influenza vaccine availability.

Question	Yes	No
Do you know that the influenza vaccine is available in Saudi Arabia for free?	316 (94.6%)	18 (5.4%)
Have you ever heard about the influenza vaccine awareness campaigns that are provided by the Saudi Ministry of Health?	301 (90.1%)	33 (9.9%)
Do you trust the information given to you by the Saudi Ministry of Health?	323 (96.7%)	11 (3.3%)
Do you trust the information given to you by your doctor?	320 (95.8%)	14 (4.2%)

**Figure 2:**
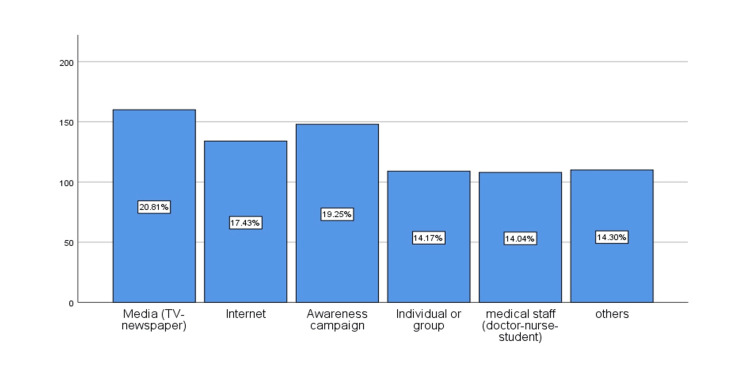
The sources of information regarding the influenza vaccine.

Figure 2 shows that participants learned about the influenza vaccine from a variety of sources, with the media, the internet, and awareness campaigns serving as the primary sources for nearly two-thirds of the participants.

## Discussion

In children, particularly those in preschool and primary school, influenza infection is significantly associated with morbidity [7]. One of the most effective methods to prevent and control influenza epidemics is an annual vaccine. The majority of influenza vaccinations effectively stimulate antiviral antibody responses [8]. In this study, we assessed the level of knowledge among Saudi parents in the Makkah region about the influenza vaccine and the barriers to and willingness to vaccinate their children.

We found that the majority of the participants were female, had a higher level of education, and were unemployed. Moreover, most of them reported that their children were up-to-date on vaccinations. Similar findings were reported by Alolayan et al., who conducted a similar study among the Saudi population [9].

Regarding prior influenza vaccination among respondents and their children, we found in our study that most of the parents and their children received it. However, we found a correlation between a higher level of education and female gender as common findings in those who did not take the influenza vaccine. In terms of the high proportion of participants who took the vaccine, this is common even in other parts of the world. It is a good thing that most people are willing to receive them. A similar finding was reported in a study from Georgia. Gargano et al. conducted a study among the parents of adolescents in Georgia, and they found that more than half of them received the influenza vaccine prior to the fall or winter [10].

When it comes to the barriers to receiving the influenza vaccine, we found that in our study, false beliefs about the mechanism of action of the vaccine as well as wrong ideas about the vaccine's adverse effects were the main barriers. In contrast, some international data showed that the main barrier to getting the influenza vaccine and for families to provide it to their children was indeed the cost of the vaccine. This was demonstrated in a Japanese study. Other international data showed parents’ concern about the safety of the vaccine and its necessity as a major barrier [11-13].

Most parents, regardless of their level of education, have poor knowledge about the influenza vaccine. These findings and the lack of proper knowledge may have an impact on receiving the vaccine. In our sample, 94.6% of the parents were aware that the influenza vaccine is available and free in Saudi Arabia.

Although the majority of participants in this study trust the Saudi Ministry of Health’s information and recommendations to give influenza vaccine to all children from six months to 18 years, many of them are still reluctant. This is in line with a previous study that found that about 90% of survey participants in Riyadh, Saudi Arabia, believe in their doctors' advice and have confidence in the Saudi Ministry of Health [14].

We think there is a gap in the education of the public with regard to the influenza vaccine. We think we need more public influenza vaccine campaigns. There are different ways in which knowledge about the vaccine can be delivered. A variety of resources can provide information regarding influenza vaccines. In other studies, it was shown that awareness campaigns, social media outlets, newspapers, magazines, television, and the internet (websites) are important tools in impacting parents' knowledge about the influenza vaccine [10, 15].

This study has some limitations. Recall bias might result from the cross-sectional design. Moreover, because the study was conducted online, the surveyor could not have assisted any participants who were having trouble answering the questions.

## Conclusions

The majority of parents, even those who have a high level of education, have a poor understanding and knowledge of the influenza vaccine. They do not realize the importance of such a vaccine. Although a large number of parents believe that they and their children are healthy and do not need the influenza vaccine, as we know, any public mass vaccination will not be successful unless it is widely implemented. So we have to do more to convince them.

This study highlights the need to increase awareness, educate parents in the Makkah region about the importance of the influenza vaccine, and encourage them to vaccinate their children. So we recommend that pediatricians, primary care physicians, and other members of the health care team do their best to advocate for the vaccine and encourage parents to get it for their children.
